# Testing modes of computerized sepsis alert notification delivery systems

**DOI:** 10.1186/s12911-016-0396-y

**Published:** 2016-12-09

**Authors:** Mikhail A. Dziadzko, Andrew M. Harrison, Ing C. Tiong, Brian W. Pickering, Pablo Moreno Franco, Vitaly Herasevich

**Affiliations:** 1 Department of Anesthesiology, Mayo Clinic, 200 First St SW, Rochester, MN 55905 USA; 2 Medical Scientist Training Program, Mayo Clinic, Rochester, Minnesota USA; 3 Department of Information Technology, Mayo Clinic, Rochester, Minnesota USA; 4 Division of Critical Care Medicine, Mayo Clinic, Jacksonville, Florida USA

**Keywords:** Methods of alert delivery, Sepsis, Alert fatigue, Notification, Decision support system

## Abstract

**Background:**

The number of electronic health record (EHR)-based notifications continues to rise. One common method to deliver urgent and emergent notifications (alerts) is paging. Despite of wide presence of smartphones, the use of these devices for secure alerting remains a relatively new phenomenon.

**Methods:**

We compared three methods of alert delivery (pagers, EHR-based notifications, and smartphones) to determine the best method of urgent alerting in the intensive care unit (ICU) setting. ICU clinicians received randomized automated sepsis alerts: pager, EHR-based notification, or a personal smartphone/tablet device. Time to notification acknowledgement, fatigue measurement, and user preferences (structured survey) were studied.

**Results:**

Twenty three clinicians participated over the course of 3 months. A total of 48 randomized sepsis alerts were generated for 46 unique patients. Although all alerts were acknowledged, the primary outcome was confounded by technical failure of alert delivery in the smartphone/tablet arm. Median time to acknowledgment of urgent alerts was shorter by pager (102 mins) than EHR (169 mins). Secondary outcomes of fatigue measurement and user preference did not demonstrate significant differences between these notification delivery study arms.

**Conclusions:**

Technical failure of secure smartphone/tablet alert delivery presents a barrier to testing the optimal method of urgent alert delivery in the ICU setting. Results from fatigue evaluation and user preferences for alert delivery methods were similar in all arms. Further investigation is thus necessary to understand human and technical barriers to implementation of commonplace modern technology in the hospital setting.

## Background

Generation of computerized notifications and alarms by automated detection algorithms for a variety of clinical conditions leads to alarm fatigue, which is one of the most important health technology hazards over past years [1]. Several solutions have been proposed to improve alarm detection conditions in the healthcare setting [2]. In the case of alarm system management, the challenge is to deliver the correct alarm, using the right alarm delivery mode, to the right recipient(s) [3].

Minimizing the number of clinically insignificant alarms (better pattern recognition), optimizing alarm notification, and response protocols are the goals to address clinical alarm hazards to insure patients receive appropriate care at the time it is needed [1, 4].

Perception of different methods of alert delivery is significantly influenced by complex human cognition factors [5, 6]. In the specific context of the hospital setting, mechanisms of notification or alert delivery have been exploited, including oral communication, charts, loudspeaker alerts, phones, paging, electronic health record (EHR) display, and email [7]. New methods of alert delivery have also led to the development of technology to reduce errors in the hospital setting [8]. [9]

Successful EHR notification delivery has the potential to reduce errors in the hospital setting [9–12]. However, it is not sufficient for a clinical alert system to be merely capable of generating medically meaningful alerts [4, 13]. This is because implementation of any automated notification or alert system must be performed in the context of information overload and complex task interruption. In ICU setting, even meaningful alerts pose the risk of interruption [14] and information overload can alter alert perception [15, 16]. There is a need to consider how a system can generate clinically meaningful alerts, while concurrently minimizing information overload and task interruption. To do this, a better understanding of human cognition and user interfaces is required [17, 18]. An ideal communication tool should enable bi-directional, rapid, secure, and non-disruptive transmission of content-rich messages [19]. It should provide specific mechanisms to avoid any potential for protected health information security breach. Knowledge of optimal methods of delivery of urgent alerts in the intensive care unit (ICU) setting, particularly with the goal to shorten time-to-reaction and to decrease alert fatigue, is limited and contradictory [20, 21].

The objective of this study was to compare three methods of alert delivery - pagers, EMR-based notifications, and smartphones - to determine the best method of urgent alerting in the ICU setting.

## Methods

### Study design and setting

This prospective randomized study was performed from October 2015 through December 2015 in the 54 beds ICU at Mayo Clinic in Jacksonville, FL. This ICU includes multidisciplinary beds including medical, surgical, transplant, and neurology critical care services. The Mayo Clinic Institutional Review Board (IRB) has approved this study. There were no changes in usual protocols of patient care. Oral consent was obtained from participating clinicians.

### Participants

Clinicians with a personal iPhone Operating System (iOS) device (smartphone or tablet) were eligible to participate. One ICU 12-hour shift was treated as a study period for each participant. Participants were already familiar with the EHR electronic sepsis alerts and SSC (Surviving Sepsis Campaign) recommendations [22, 23] as they were routinely used in the ICU.

### Alert generation

The automated, EMR-based sepsis detection tool (sepsis sniffer), already validated and described elsewhere [24], continuously assessed EHR data for sepsis criteria. In a case of automated detection, it generated an alert - a yellow triangular icon appeared within the EHR, indicating individual patient with sepsis. Standard practice includes alerting a nursing team leader by a pager, who makes decision to activate a rapid response team (RRT). For the purposes of the study generated alerts were sent to participants using randomly selected delivery method. These alerts included the following text: “Shock alert: sepsis detected, room XXX”.

### Alert delivery and randomization algorithms

**Fig. 1:**
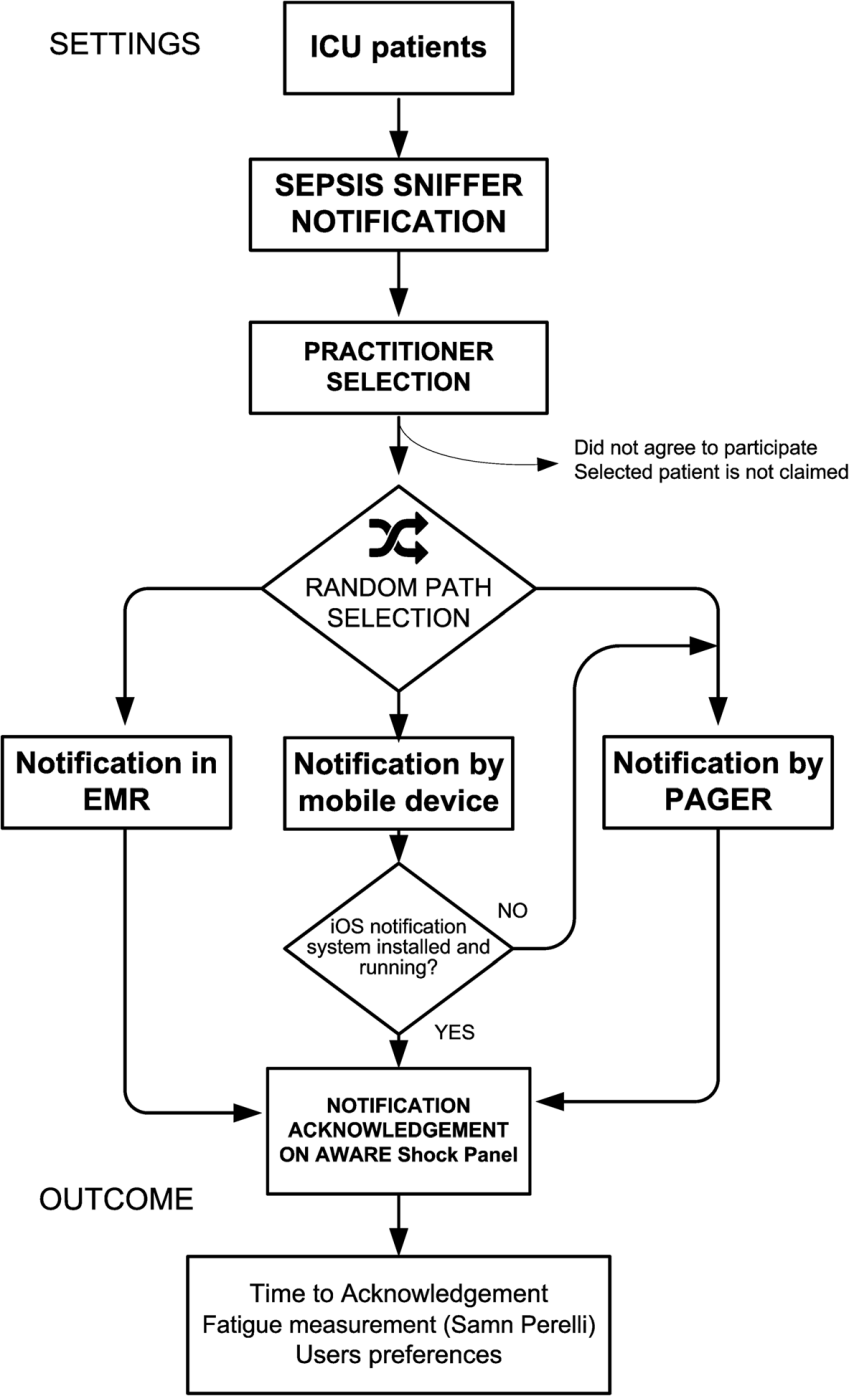
The study workflow for alert delivery. In a case of a negative check for the iOS arm eligibility, selected alerts were redirected to the pager arm. EMR, electronic medical record; ICU, Intensive Care Unit; iOS, iPhone Operating System

Three methods of alert delivery were used: pager, EHR-based monitor display notification, and iOS-based smartphone/tablet (Fig. 1).

At the beginning of study periods, participating clinicians manually triggered a specific flag within the EHR to “claim” hospitalized patients and become an “assigned practitioner”. This flag cleared automatically at the end of each study period. If there was no assigned clinician, as a safety net, an alert was rerouted following standard practice. At the time an alert is generated, an algorithm randomly determines which of 3 routes will be used to deliver that alert to the clinician who has 'claimed' that patient. Participating clinicians could receive more than one alert during one study period.

### iOS device pathway description

Sepsis alerts were delivered using a standard practice iOS application at Mayo Clinic (Synthesis Mobile) complied with protected health information (PHI) encryption and HIPAA specifications [25]. This application uses a secured virtual private network (VPN) connection and allows delivery of pop-up notifications. The following conditions were required to exploit iOS device pathway: a) the patient must be ‘claimed’, b) Synthesis Mobile must be running on person’s device, c) VPN connection must be established. Each participant was given instructions before the beginning of the study to ensure that latest Synthesis Mobile Application was installed on their device and how to make sure that the application was running on the background during their shifts. In the case of failure to meet all criteria of eligibility, alerts assigned by the randomization algorithm to iOS were sent by pager instead. Prior to study implementation, notification system was successfully piloted on pagers, the EHR, and iOS devices.

### Outcomes measurements

Alert delivery was acknowledged by clinician participants through the EHR using individual personal credentials by entering login and password. The primary outcome was time to acknowledgment, which was defined as the difference between time of alert generation and the time of interaction with the alert in the EHR.

The secondary outcome was time from alert generation to SSC 3-hours bundle completion (‘time-to-goal’) [22, 23, 26]. Also measured were time from acknowledgement to time-to-goal, and timeliness of acknowledgment when time-to-goal was achieved. We assessed clinician participant experience with methods of alert delivery, using a 7-points Likert-like scale from extremely dissatisfied (1) to extremely satisfied (7). A post-shift fatigue was evaluated using the validated 7-points Samn-Perelli (SP) scale scoring fatigue from “1 - fully alert, wide awake” to “7 - completely exhausted, unable to function effectively” [27, 28].

### Data handling and statistical analysis

For the power calculation we used mean time to acknowledgement of 13 minutes, based on the study of Moss and colleagues (median response 3 mins, IRQ 1-8 mins, and less than 15 mins for ‘adequate’ response) [29]. For mean time to acknowledgement of 13 mins, with a presumed standard deviation of 7 mins, 240 measurements (alerts) should produce 84% power. The number of unique patients and number of alerts per unique patient directly influence the number of potential clinician participant responses. However, these factors are independent of the power calculation of the number of clinician participant measurements needed.

Data collected by the randomization algorithm was stored in a dedicated table within an institutional research warehouse database. An online electronic survey was conducted at the end of study to measure clinician participant experience and fatigue results. Continuous variables are presented as median ± interquartile range. Categorical variables are presented as count and percentage. Multiple statistical comparisons were performed using the Kruskal-Wallis or chi-square test as appropriate. A two-tailed p-value of less than 0.05 was considered statistically significant. Statistical software JMP 11 (SAS, Cary, NC) was used for all calculations.

## Results

Out of 40 potentially eligible clinicians, 23 (58%) agreed to participate in this study. All clinician participants had iOS devices (smartphone or tablet). During the 3 month study period using this sepsis-detection decision algorithm, there were 69 alerts in 67 patients. However, only 46 patients were “claimed”, and a total of 48 alerts were sent by the randomization algorithm (Table 1). All of these alerts were acknowledged.

**Table 1:** Primary and secondary outcome results

	Total	EHR	Pager	iOS	*P* value
Number of alerts generated	48	14	12	22	-
Number of alerts sent	48	14	34	0	-
N of participant received alert	23	12	11	-	-
Time to acknowledgment, min (minimal-maximal)		169 [71-348] (14-538)	102 [6-288] (<1-2066)	-	0.15
Number of patients who achieved SSC bundle goals		5	16	7^a^	
Time to achieve goal, min		159 [125 - 200]	164 [140-176]	-	0.5
Time from acknowledgement to achieve goal, min^b^		13 [-235-131]	70 [-129-146]	-	0.48
Timeliness of Acknowledgment^c^		Timely 60%	Timely 56%	-	0.9
Experience with notification method		4 [3, 4]	4 [3-5]	-	n/s
SP fatigue score		4 [3-5]	4 [3-5]	-	n/s

^a^Alerts from 7 patients randomized to the iOS arm were redirected to the pager arm

^b^Negative values indicate the alert was acknowledged after completion of the SSC bundle

^c^Timeliness of Acknowledgement indicates acknowledgement of sepsis alerts in the EHR before completion of the SSC bundle

EHR, electronic health record; iOS, iPhone Operating System; SP, Samn-Perelli; SSC, Surviving Sepsis Campaign

The study was terminated prematurely because of failure of the iOS arm. All patients in the iOS arm (*n* = 22) were claimed and participating clinicians was assigned, but the algorithm of alert delivery failed to detect the presence of the dedicated iOS EHR application in all of these cases. This caused all alerts assigned to this arm to be rerouted to the pager arm. The median time to acknowledgement for EHR-embedded notifications was 170 minutes (*N* = 14) and 102 minutes (*N* = 34) for alerts delivered by pager (*p* = 0.15). The SSC bundle was completed for 60% (*N* = 28) patients. In the case of these patients, the median time from alert generation to SSC bundle completion was 159 minutes for EHR-notifications and 164 minutes for pager notifications. At the end of the study participants were satisfied with the both methods of alert delivery. The median fatigue score at the end of shifts was equal in both arms.

## Discussion

Methods of urgent alert delivery in the ICU setting were evaluated in this randomized prospective study. The study was terminated before target accrual was reached because the iOS randomized arm was not able to deliver alerts from the sepsis sniffer. Time to acknowledgment was almost 2 hours for the pager arm and 2.5 hours for the EHR arm, which was not a statistically significant difference. For patients in both arms for whom the SSC bundle was completed, 60% of alerts were acknowledged before bundle goals. However, this time likely does not represent the time of alert reception by clinicians. The experience from the survey about EHR and pager methods of notification delivery was neutral to positive in both groups. No difference in Samn-Perelli fatigue score was detected between participants in the assigned arms.

Traditional computerized paging system notifications are widely implemented and frequently used. The content of delivered information is limited to a finite number of characters and it is also not possible to send immediate feedback to the sender. Urgent character of alerts does not force clinicians to take immediate action in the EHR. The provider often prioritizes his attention to the patient until the initial goals of care are reached, and thereafter acknowledge the alert in the EHR. Although this EHR login step was important in this study setting to acknowledge alert receipt, it was seemingly not used routinely by clinician participants. This can explain the important lag time between alert generation and alert acknowledgment in the pager arm.

Standard text messaging is discouraged for transfer of confidential information in the healthcare setting due to lack of HIPAA compliance. Advanced two-way systems, such as cellphone or smartphone text message, save time, increase efficiency, facilitate better patient care, decrease callbacks, and reduce interruptions of educational activities [30].

Smartphones and tablets were found to be a reliable method to deliver time-critical information. However, transferred and encrypted PHI can be viewed by the host server [25, 31] which can lead to HIPAA violation. A secure, VPN-based connection was used in this study, enabling encrypted HIPAA-compliant transmission of PHI. A VPN connection had to be established prior the launch of the Application. However, of 22 correctly assigned alerts, none of them reached the designated clinician because the algorithm was not able to detect an established VPN connection or a running specific application on the iOS device at time of alert. At our institution a VPN connection establishes automatically, it usually only requires to be set up once and then upon entering the building credentials are automatically authenticated. This points our attention to the lack of running application as the most plausible culprit. Several possibilities would explain lack of running application include: failure to install the lasted application, failure to configure their application to allow notifications and failure to launch the application at the beginning of their shift. In a busy clinical environment adding one more step like launching an application during hand-off communication may get overlooked.

Personal mobile devices in the professional settings are used mostly for non-urgent and less time-sensitive purposes and are not considered as primary messengers. One concern that would remain would be the competing priorities of other personal alerts, email, texts or pop-ups from personal business. Recent study [21] has been shown that mobile device notifications were disruptive and negatively influenced on the performance during attention-demanding task, even when participants did not directly interacted with a mobile device during the task.

Timely acknowledgement of EHR-embedded notifications requires constant physical proximity and the use of personal credentials to log in to the system, or again the use of VPN connections through portable devices. EHR-embedded alerts were studied using computerized prescription order entry (CPOE) systems. A median lag time for the interrupting alerts for drug-drug interactions was 8 seconds and varied from 1 second to 34 mins [32]. In our study for EHR embedded alerts the minimal time to acknowledgment was 14 mins and maximal 34 hours (for one participant). Theoretically any alert sent within an EHR has an instant delivery, and can have a reliable feedback. This is one of strengths of this type of delivery method. As the accuracy of electronically generated alerts is imperfect, the feedback loop for any clinical notifications can help not only to ameliorate quality of care, but also to optimize and diminish alert fatigue [33, 34]. However, working stations with EHR-embedded notifications were inappropriate to display time critical notification messages in the ICU settings [20] due to limited duration of login authentication intervals, which delayed acknowledgements. ICU providers may not have the ability to monitor the EHR for new alerts, so in this scenario having the pager system seems to be the most reliable from studied methods.

Pager devices remain a gold standard for urgent and non-urgent notifications. Reported use of this highly reliable technology is about 90% out of 200 hospitals [35]. However, the cost of pager mediated alert delivery method seems to be almost 2 times higher than the cost of secure messaging apps.

The urgency and prioritization of transmission using different methods of notification delivery is important to address in the scope of the phenomenon of alert fatigue. However, this study was not able to detect any difference in the fatigue of clinician participants. This was partly the result of a small number of observations and lack of validated methodology to assess alert fatigue in the ICU setting.

This study has several limitations. (1) This was a single-center study at an academic medical center. Well-established biases and potential confounders are known to be present with this particular study design. (2) Simple randomization without blocking was used, which may have led to unequal allocation due to small sample size. (3) HIPAA compliant mechanisms for iOS-based mobile devices are institution-specific and have led to technical failure. Thus, this aspect of the results of this study may not be completely generalizable. (4) Due to iOS arm failure the study was underpowered. (5) The Samn-Perelli scale is a subjective tool and thus may not be sensitive enough to fully address all aspects of alert fatigue. (6) Time to acknowledgement as it was designed, probably does not represent the time when clinicians became aware of the alert, due to the human-factors barriers to implementation of alert studies in the clinical setting. This phenomenon was also explored in a parallel study in the ICU setting (Harrison AM, Thongprayoon C, Aakre C, Jeng J, Dziadzko MA, Gajic O, Pickering B, Herasevich V: Barriers to implementation of an automated severe sepsis alert system in the ICU setting, submitted).

## Conclusions

This study did not determine the optimal method of urgent alerts in the ICU settings using HIPAA compliant transmission protocol. Personal iOS-based devices were not reliable tools to deliver alerts using HIPAA compliant methods due to specific network connection requirements. Further investigation is thus necessary to understand human and technical barriers to implementation of prominent wireless electronic technology in the healthcare setting.

## Abbreviations

CPOE: Computerized physician order entry
EHR: Electronic health record
HIPAA: Health Insurance Portability and Accountability Act
ICU: Intensive care unit
iOS: iPhone operative system
IQR: Interquartile range
IRB: Institutional review board
PHI: Protected health information
RRT: Rapid response team
SSC: Sepsis survival campaign
VPN: Virtual private network
